# A Cross-Sectional Study of Smoking Behaviors and Attitudes of Parents in Pediatric Primary Care Settings

**DOI:** 10.3390/ijerph15071384

**Published:** 2018-07-02

**Authors:** Aleksandra Ratajczak, Karol Ratajczak, Wojciech Feleszko

**Affiliations:** Department of Pediatric Respiratory Diseases and Allergy, Medical University of Warsaw, Żwirki i Wigury 63A, PL-02-091 Warsaw, Poland; olarurarz@gmail.com (A.R.); karolman890@gmail.com (K.R.)

**Keywords:** tobacco, environmental tobacco smoke exposure, children, ETS, smoking, pediatrics, family doctor, parent, smoking cessation, tobacco control

## Abstract

Environmental tobacco smoke (ETS) exposure is considered an important public health issue in pediatric population. In this study, we aimed to investigate parents’ knowledge on side effects of passive smoking and counseling for parental smoking among pediatricians and family practitioners. Participants were biological parents of pediatric patients up to the age of 18 years old who attended Pediatric Hospital of Medical University of Warsaw. The questionnaire included 28 questions and queries on environmental tobacco smoke in children’s environment. Medical students identified potential subjects and handed out previously created questionnaires. In total, 506 parents of children aged 0–18 years old were interviewed; 41% (207/506) of parents were smokers, 23% (114/506) were asked about ETS exposure by their pediatricians and 41% (205/506) by family physicians during routine visits. Only a minority of the respondents confirmed having “no smoking” policy in their car 31% (157/506) or in their households 24% (121/506). All parents believed that passive smoking could cause at least one harmful effect: most common were more frequent respiratory infections (43%), asthma (40%), and low birth weight (37%). Among smoking parents, 38% (78/207) has tried to quit smoking for their child’s health sake; 63% (131/207) of smokers have never been asked to quit smoking by their doctor. Parents’ understanding of passive smoking among children differs from current medical knowledge. Rates of screening and counseling for parental smoking in pediatric and family practices are still unsatisfactory.

## 1. Introduction

Despite public awareness of the dangers of environmental tobacco smoke (ETS) among children, approximately half of the world’s population of children is constantly exposed to passive smoking [1,2,3]. Large differences in smoking prevalence exist between countries and in different environments (i.e., urban vs. rural, socioeconomic classes). In the United States almost 43% of children aged from 2 months to 11 years of life live in a home with at least one person who is a smoker [4]. The prevalence of passive infant smoking was reported to be around 40% also in Europe [5].

Tobacco smoke inhaled during passive smoking is a mixture of gases and microparticles containing nicotine and different toxic substances, irritants, and carcinogens [2,6]. In comparison to adults, children have a higher frequency of breaths per minute, their hepatic metabolism is not yet fully developed and they inhale more air per body weight which explains why children are more susceptible to tobacco smoke exposure [7]. It has been demonstrated that children’s exposure to ETS is associated with sudden infant death syndrome, low birth weight (less than 2500 g), recurrent respiratory tract inflammations, and contributes to the development of asthma [2,8,9]. Nonetheless, a significant number of parents do not realize the harmful effects of smoking on the health of their children. For this reason, it is considered that parents should receive comprehensive information about the negative impact of passive smoking on the health of their children. In our preliminary study, we assessed the role of pediatricians and family doctors in initiating smoke-free interventions in smokers’ parents [10]. The aim of our current research is to determine parents’ knowledge about the side effects of passive smoking among children and to evaluate and compare the effectiveness of pediatricians and family doctors in the field of screening and anti-smoking counseling.

## 2. Materials and Methods

### 2.1. Sample and Settings

Participants were biological parents of pediatric patients up to the age of 18 years old who were attending the departments of pediatrics in Pediatric Hospital of Medical University of Warsaw.

### 2.2. Procedures

Participants were recruited, during September 2016–January 2017, through medical students who participated in the protocol development workshop of the project in an earlier stage. Students were provided with the verbal and written background information of the study and the characteristics of people we were looking for to participate in our study. Selection criteria were as follows: father or mother of a pediatric patient, age > 18, smoker or nonsmoker or former smoker, willing to give consent to participate in our study. Students identified potential subjects and handed out previously created questionnaires. We conveniently asked five to six interested parents to fill in our questionnaires and we were available for more information for parents in the room where they filled out questionnaires. The study was carried out following the rules of the Declaration of Helsinki of 1975, blinded to study individuals and informed consent from all responders was obtained.

### 2.3. Data Collection

A questionnaire was developed with reference to the research team’s earlier work and was created according to the one, used by Rosen, et al. [11] (Appendix A).

The questionnaire was then pilot tested with three parents resulting in minor changes. All of the questionnaires were conducted by writing down answers by parents. The questionnaire included 28 questions and queries on the following themes: risks of smoking and ETS exposure, attitudes towards ETS, situations where children are exposed to ETS, experiences with the child’s pediatrician or family doctor about smoking or ETS exposure, and views about pediatrician engagement in promoting ETS exposure reduction and parental smoking cessation. Two medical students conducted all the interviews. Interviewers were medical students at the Medical University of Warsaw who attended a three-hour training course on qualitative research methods and tobacco use reduction research. The training also included a session on the ethical aspects of human subject research. To collect data, two interviewers worked as a team-one handed out the questionnaires and the other assisted parents with answering questions that posed any trouble. All questionnaires were filled out at the hospital in a meeting room for patients and it lasted for approximately 10 min. The sessions started with the moderator explaining the purpose of the research and assuring confidentiality of the data collected for the research project.

### 2.4. Analyses

The interviewers summarized the content of each questionnaire by the use of Microsoft Excel 2016 version 16.0.6741.2048. All additional notes written on questionnaires by parents were examined to identify various themes presented in these qualitative discussions and to understand the main concern about ETS parents have. All unclear answers, unfinished questions, or logistic errors, were rejected.

## 3. Results

**Figure 1:**
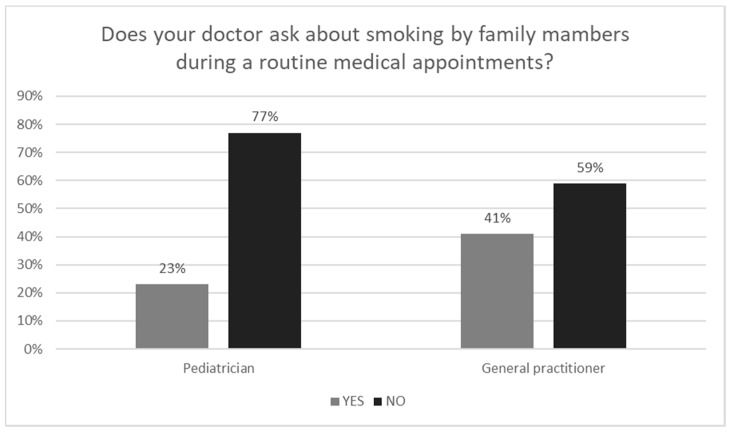
Screening and counseling for parental smoking in primary health care settings.

**Figure 2:**
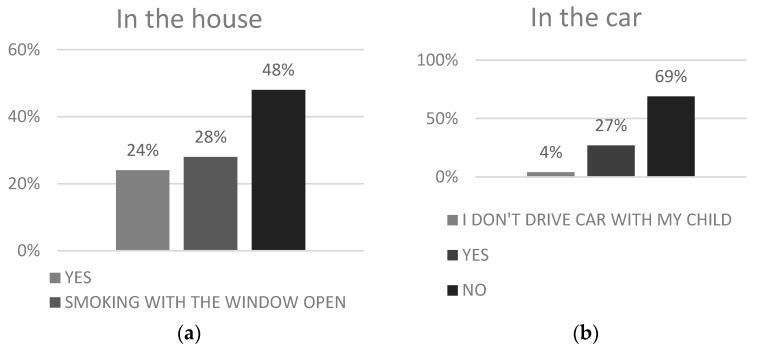
No smoking policy in child’s environment: (**a**) in the house; (**b**) in the car.

**Figure 3:**
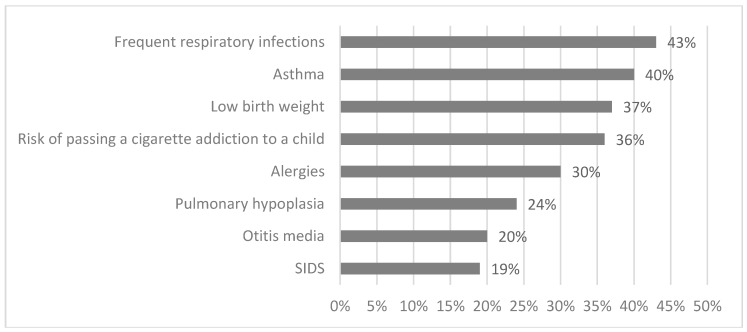
Negative health effects of SHS exposure among children-parental beliefs.

In our cross-sectional study, 59% (299/506) of parents denied smoking, the remaining 41% (207/506) claimed to be active smokers. We have conducted an analysis of different socio-demographic factors (i.e., place of caregiver’s birth, educational level, and age of respondents) and the results were inconclusive as no significant differences were observed among various social classes. Reportedly, only 23% (114/506) of parents claimed, that during pediatric appointment with their child the issue of tobacco smoking among family members was raised (Figure 1). Adversely, as much as 59% (301/506) of family physicians inquired parents about dependence on cigarettes and their smoking habits. Nearly 69% of respondents (349/506) had no rule banning smoking cigarettes in the vehicle used by their child. The no smoking in the house rule only applied to every fourth household (24% of respondents declared to find a use of this regulation in their house) (Figure 2). Every respondent (100% participants) believed that passive smoking causes at least one negative effect on the health of their child. Detailed analysis revealed, that the most common fears raised by responding caregivers were: frequent respiratory infections (43%), asthma (40%), low birth weight (37%), risk of passing a cigarette addiction to a child (36%), allergies (30%), pulmonary hypoplasia (24%), otitis media (20%), sudden infant death syndrome-SIDS (19%), and others such as headache, emphysema, problems with concentration, cough, and disturbance of smell and taste (Figure 3). 38% (78/207) of smoking parents have tried to quit smoking, and the most common motivation was the health of their child. 25% of parents (99/506) would not have objected if their child had become an active smoker. It is disturbing that as many as 63% (131/207) of surveyed caregivers who smoke cigarettes have never had anti-smoking therapy proposed by their pediatrician or family doctor.

## 4. Discussion

Our study has illustrated low rates of screening and counseling for parental smoking among pediatricians and family doctors in Poland. Only half of all parents surveyed were screened for the presence of household smokers and only about one third of parental smokers were counseled about the dangers of child tobacco smoke exposure or the risks associated with ETS for children. The low rates of smoking prohibitions inside home (24%) or car (31%) are exceptionally disturbing, given the child morbidity and mortality associated with ETS. Recommendations from the American Academy of Pediatrics and the American Academy of Family Physicians clearly highlight the significance of screening and counseling activities during primary health care visits regarding children [12,13].

We would like to draw attention to the discouraging aspect of the effectiveness of anti-smoking interventions during routine visits at the pediatrician. According to a study conducted by Deborah et al. in the United States, the vast majority of parents (95%) believe that the aspect of cigarette smoking by any family member is a significant part of the medical history at the child’s pediatrician’s office [14]. However, the correlation between parental smoking and recurrent respiratory infections—as well as a higher incidence of asthma—appear to be still underestimated by most pediatricians and general practitioners. Considerable discrepancies between different cultures and nations in the field of screening of passive smoking among children are observed. Our research has shown that in Poland 77% pediatricians do not ask about smoking by family members during routine medical appointments. For comparison, in the United States only 27% of pediatricians do not ask parents about dependence on cigarettes and habits associated with it [15], while in Israel, the rate of medical doctors who do not collect data about smoking from their patients remains at 43%. The statistics shown above indicate relatively low efficiency of pediatricians in the field of screening and anti-smoking counseling.

According to our research, a rule banning smoking in the car in which a child travels was valid for only 23% of Polish families. It is crucial to underline that in Poland there is no regulation restricting personal behavior in a vehicle one owns. This result is explicitly unsatisfactory compared with other countries. In Australia, in a similar study concerning parental personal behavior during traveling in a car with a child, up to 96% of respondents claim that they prohibit smoking in the car [16]. It correlates however with the fact that a ban on smoking in cars carrying children (aged under 18) became effective in most of Australian states. Correspondingly, in a complementary Welsh study, only 9% of the respondents agree for smoking inside the vehicle, which is used by the child [17]. A ban on smoking in cars carrying children under 18 came into force in Wales in October 2015. Taken all together, these results imply that legislative changes may play a key role to help reduce ETS exposure among children.

Despite the fact that children ordinarily spend less time exposed to ETS in the car than at home, a small and closed nature of the vehicle makes this tobacco smoke exposure very intense [7]. It is therefore crucial that pediatricians and general practitioners indicate an urgent need to quit smoking in vehicles used by children during every medical appointment. Murphy-Hoefer, et al. has demonstrated the beneficial effects of ‘no smoking in the car’ regulation. In their study, they have discovered that smokers’ parents who implemented a ban on smoking in the car were afterwards considerably more successful in smoking cessation [18]. Therefore, a rising number of countries—including Australia, Canada, and the United States—have introduced bans on smoking in cars where children are passengers [17,19,20].

Additionally, various studies imply that tobacco-free work and public areas and the likelihood of the no-smoking rule implementation in the smoking parents households are closely related [21,22,23,24,25]. Public health strategies are required to reduce ETS exposure at home among children.

We do agree with the hypothesis of Daly et al. that the current method of smoking cessation interventions are not sufficient, perhaps due to the lack of attractive tools for smoking cessation [26]. E-cigarettes seem to be an interesting alternative, regarding great interest of the public, and recent data suggesting that, even if they are not completely neutral on human health, they are probably less harmful for children than conventional cigarettes [27].

It needs to be mentioned that children are exposed to ETS not only during unintentional inhalation of side stream tobacco smoke from an active smoker. The combination of tobacco smoke pollutants which remain in an indoor environment, which is referred to as ‘third-hand smoke’ (THS) causes unintentional ETS intake [28]. THS consists of noxious substances that remain on surfaces and in dust after tobacco has been smoked, and are re-emitted into the gas-phase, or react with other compounds in the environment to form secondary contaminants that are toxic for humans [29]. Exposure to THS may take place long after the cessation of active smoking in indoor environment in which cigarettes are regularly smoked. THS represents a new concept in the field of tobacco control and should be considered as an individual aspect of passive smoking exposure.

This study has several limitations. To access parental smoking behavior we used data based on a questionnaire obtained from a family member. It is possible that parents or caregivers involved in this study underestimated their child’s tobacco exposure. It is also probable that respondents answered what they believed to be the ‘right’ choice (i.e., social desirability bias occurred). Secondly, smoking variables were self-reported without biomarker validation which may pose a risk of belittled answers regarding ETS exposure risk behaviors. However, the reliability of self-reported smoking behavior is generally high [30]. In our study, we relied on parental report of primary health care services. This may not be an accurate assessment of what has genuinely occurred, as it is may be presumed that parents forget or misremember details of their child’s physician visit over a period of time.

Despite these limitations, this study has some strengths of a representative sample with possible generalizability for the whole country and a prospective design that allows examination of a detailed picture for parental smoking cessation reasons with multiple covariate adjustments.

Future research using biophysical measures of ETS exposure among children is needed. It is necessary to establish parental underestimation of the impact of ETS exposure on their children. Efforts should now focus on determining the best screening and counseling methods for parental smoking in the child’s primary health care system in a consistent and effective manner.

## 5. Conclusions

Screening and counseling for parental smoking by primary health care services is insufficient. It would be beneficial for children’s health if pediatric primary care settings improved their performance in the area of tobacco smoke control as exposure to ETS has adverse effects on child’s health.

Smoking in public places was banned in Poland on 15 November 2010 by a change in parliamentary act “On Defending Health against Results of Tobacco and Tobacco Products Usage”. Smoking is prohibited now in schools, hospitals, or other medical facilities and public transport (including the vehicles such as train or bus and bus stops, train stations, etc. within a 10-m radius).

In March 2010, an attempt to introduce a complete smokefree law failed. There was a wide debate on prohibiting smoking in vehicles with children but this amendment did not pass due to public opinions that this was a violation of rights and right to private property. Preventive actions are not only raising the awareness of parents and primary health care system’s doctors but they also provoke changes in legislation. Therefore, it is necessary to focus on finding an attractive and effective approach to implement screening in routine pediatric services.

## Figures and Tables

**Figure A1:**
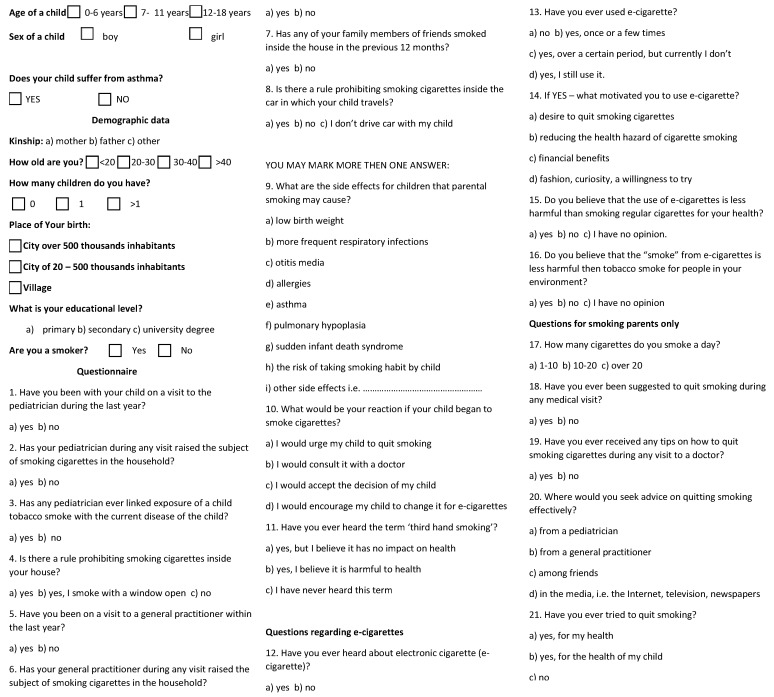
Questionnaire for parents regarding cigarette smoking and SHS exposure among their children.
